# Ab Initio Studies of Work Function Changes Induced by Single and Co-Adsorption of NO, CO, CO_2_, NO_2_, H_2_S, and O_3_ on ZnGa_2_O_4_(111) Surface for Gas Sensor Applications

**DOI:** 10.3390/s26020415

**Published:** 2026-01-08

**Authors:** Jen-Chuan Tung, Guan-Yu Chen, Chao-Cheng Shen, Po-Liang Liu

**Affiliations:** 1 Center for General Education, Chang Gung University, Taoyuan 33302, Taiwan; jctung@mail.cgu.edu.tw; 2 Graduate Institute of Precision Engineering, National Chung Hsing University, Taichung 40227, Taiwan; g7112067012@smail.nchu.edu.tw (G.-Y.C.); 7113067003@smail.nchu.edu.tw (C.-C.S.); 3 Department of Applied Materials and Optoelectronic Engineering, National Chi Nan University, Nantou 54561, Taiwan

**Keywords:** gas sensor, density functional theory, work function

## Abstract

**Highlights:**

**What are the main findings?**

- Co-adsorption of O_3_ and NO_2_ on ZnGa_2_O_4_(111) significantly enhances electron transfer, leading to the most significant work function variation and adsorption energy, thereby improving gas sensor sensitivity.
- H_2_S is the only gas among those studied that decreases the work function upon adsorption on the ZnGa_2_O_4_(111) surface.

**What are the implications of the main findings?**

- When H_2_S forms a binary co-adsorption with other gases, the overall work function variation is reduced.
- These results highlight the unique role of H_2_S in modulating surface electronic properties during gas co-adsorption.

**Abstract:**

In this study, first-principles density functional theory (DFT) calculations were employed to investigate the effects of single and binary gas adsorption of NO, CO, CO_2_, NO_2_, H_2_S, and O_3_ on the ZnGa_2_O_4_(111) surface. For single-gas adsorption, O_3_ adsorbed on surface Ga sites induces a pronounced work-function increase of 0.97 eV, whereas H_2_S adsorption at surface O sites yields the strongest adsorption energy (−1.21 eV), highlighting their distinct electronic interactions with the surface. For binary co-adsorption, the NO_2_-O_3_ pair adsorbed at Ga-coordinated sites produces the largest work-function shift (1.88 eV), while adsorption at Zn sites results in the most stable configuration, with an adsorption energy reaching −3.98 eV. These results indicate that co-adsorption of highly electronegative gases can significantly enhance charge transfer and sensing response. In contrast, mixed oxidizing–reducing gas pairs, such as NO_2_-H_2_S, lead to a markedly suppressed work-function variation (−0.02 eV), suggesting reduced sensor sensitivity due to compensating charge-transfer effects. Overall, this work demonstrates that gas-sensing behavior on ZnGa_2_O_4_(111) is governed not only by individual gas–surface interactions but also by cooperative and competitive effects arising from binary co-adsorption, providing insights into realistic multi-gas sensing environments.

## 1. Introduction

Rapid industrialization has resulted in extensive emissions of exhaust gases, toxic compounds, and wastewater, posing significant environmental and health challenges. Air pollution is particularly concerning due to its transboundary nature. Primary pollutants, including CO, CO_2_, SO_2_, NO_x_, hydrocarbons, and particulates, originate from natural events, stationary sources, and mobile sources [1,2]. These pollutants can undergo atmospheric transformations to form secondary species, such as O_3_, H_2_SO_4_, HNO_3_, and fine particulate matter, which are strongly associated with respiratory and cardiovascular diseases. According to the 2019 Global Burden of Diseases study, air pollution accounts for nearly nine million premature deaths annually, with approximately 6.7 million attributable to household, environmental, and industrial sources [3]. These statistics highlight the urgent need for highly sensitive and selective environmental monitoring technologies.

Metal-oxide-semiconductor (MOS) gas sensors have attracted considerable attention for environmental monitoring, as multiple gases often coexist in real-world conditions. Binary MOS materials, including ZnO, Ga_2_O_3_, WO_3_, and SnO_2_, have been widely studied [4,5,6,7,8]. While binary MOS materials dominate current commercial gas-sensor technologies, ternary oxide systems such as ZnGa_2_O_4_ (ZGO) are still primarily explored at the research level [9,10,11,12,13]. Challenges, including long-term stability and large-scale manufacturability, remain open questions. In this context, ZGO serves as a promising model system for investigating how mixed cation chemistry and surface coordination influence gas adsorption and sensing-related electronic responses. ZGO adopts a standard cubic spinel structure (*Fd*-3*m*), in which Zn^2+^ predominantly occupies the tetrahedral (A) sites and Ga^3+^ resides in the octahedral (B) sites. A small fraction of Zn^2+^ ions may occupy octahedral B sites, forming Zn-on-Ga antisite defects and imparting partially inverse spinel characteristics. These structural features strongly influence optical and electronic properties, including near-infrared persistent luminescence [14]. Its wide bandgap (4.72–5.07 eV), high thermal stability, chemical resistance, and ultraviolet transparency make ZGO a promising oxide material for gas sensing at moderate-to-elevated temperatures, rather than in extreme environments [15]. In general, metal-oxide semiconductor sensors are designed for intermediate-temperature operation, where material stability and surface reactivity must be evaluated comparatively across different oxide systems. Under such conditions, potential issues related to reduction or long-term stability are material-dependent and are commonly observed across a wide range of metal-oxide semiconductors, rather than being specific to ZGO.

ZGO thin films grown via metal-organic chemical vapor deposition (MOCVD) exhibit stable crystal structures and controllable defect distributions, which enhance gas-sensing sensitivity and reproducibility [15,16]. X-ray diffraction (XRD) analyses confirm strong (111) orientation of ZGO thin films on *c*-plane sapphire substrates [16]. Both first-principles simulations and experiments indicate that the ZGO(111) surface possesses optimal energy stability and efficient strain relaxation. In contrast, Ga-Zn-O-terminated ZGO surfaces have lower surface energies, favoring directional growth and stable surface configurations during deposition [17,18]. These findings support the suitability of ZGO(111) as a robust sensing platform [19,20,21,22,23,24].

Surface modification and co-adsorption significantly affect gas sensing performance. Our previous study showed that Pd atom doping on ZGO(111) enhances CO, NO_2_, and H_2_S adsorption, increases work function response, and stabilizes electronic interactions [25,26]. Similarly, studies on SnO_2_ nanofilms reveal that multi-gases co-adsorption and competitive adsorption mechanisms critically influence sensor response, with adsorption sequence and surface charge modulation affecting gas selectivity and reactivity [27]. These results underscore the importance of understanding multi-gas interactions and surface electronic effects for high-performance sensor design.

The redox behavior of NO has been described inconsistently in the literature, depending strongly on the oxide material and sensing mechanism involved. Wu et al. reported that NO acts as “an extremely toxic oxidizing gas with a pungent odor” whose adsorption on ZGO thin films increases resistance by withdrawing electrons from the conduction band—thus behaving as an oxidizing gas for ZGO-based sensors [15]. In contrast, Akamatsu et al. categorized environmentally hazardous gases into oxidizing gases (e.g., NO_2_, CO_2_, Cl_2_) and reducing gases (e.g., NO, H_2_S, CO, C_2_H_5_OH), treating NO as a representative reducing gas on WO_3_/Co_3_O_4_ systems [28]. This variability indicates that the apparent role of NO can switch between oxidizing and reducing states depending on the metal oxide substrate and operating conditions.

Furthermore, CO_2_ is consistently classified as an oxidizing gas in metal-oxide semiconductor sensors. Akamatsu et al. placed CO_2_ in the oxidizing-gas category [28], while Abdelkarem et al. emphasized that CO_2_ strongly attracts electrons and thus acts as an oxidizing agent on *p*-type CuO:Ba sensing films [29]. In this study, NO is therefore regarded as a gas with dual oxidizing/reducing character, whereas CO_2_ is treated as a purely oxidizing species.

Building on these insights, the present study investigates the co-adsorption of two gases on the ZGO(111) surface and its impact on gas sensing sensitivity. The present study aims to elucidate the mechanistic basis of multi-gas interactions, including adsorption behavior, charge transfer, and work-function modulation, using first-principles simulations. The findings are expected to guide the rational design of next-generation multi-gas sensors with improved sensitivity, selectivity, and stability under complex environmental conditions.

## 2. Computational Methods

**Figure 1:**
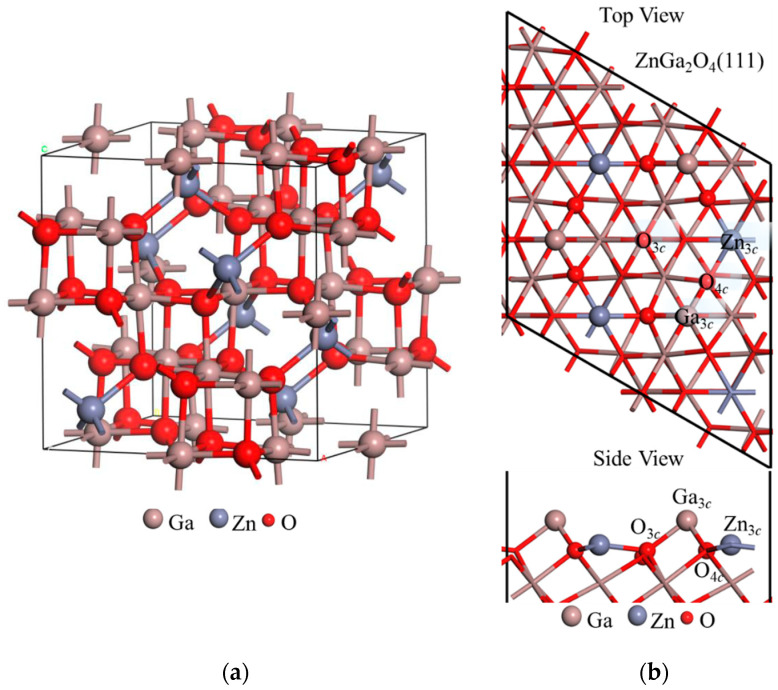
(**a**) ZGO is a spinel-structured material in which Ga atoms predominantly occupy octahedral sites coordinated by six oxygen atoms. In comparison, Zn atoms reside at tetrahedral sites coordinated by four oxygen atoms; (**b**) The structure of the ZGO(111) surface is illustrated in the top and side views. In these representations, brown atoms correspond to Ga, gray atoms to Zn, and red atoms to O. The ZGO(111) surface features four preferred adsorption sites, namely Ga_3*c*_, Zn_3*c*_, O_3*c*_, and O_4*c*_.

The primary focus of this study is to examine the effects of co-adsorption of NO, CO, CO_2_, NO_2_, H_2_S, and O_3_ on the work function of the ZGO(111) surface; however, the results of single-molecule adsorption are also included. This comparison provides a clearer understanding of the distinctions between single- and dual-gas adsorption behaviors on ZGO(111). In this study, systematic ab initio calculations were carried out to investigate the equilibrium bond lengths, adsorption processes, and work functions of NO, CO, CO_2_, NO_2_, H_2_S, and O_3_ molecules adsorption on ZGO(111) surfaces. All simulations were performed using the Vienna ab initio Simulation Package (VASP) [30,31] within the generalized gradient approximation (GGA) and the Perdew-Wang (PW91) correction [32,33]. The bulk ZGO crystal, shown in Figure 1a, adopts a cubic *Fd*-3*m* structure, modeled with 8 Zn, 16 Ga, and 32 O atoms per unit cell. In this structure, Zn^2+^ and Ga^3+^ cations occupy tetrahedral and octahedral lattice sites, respectively. The plane-wave cutoff energy was set to 450 eV, and the convergence criterion for the self-consistent total energy was fixed at 10^−5^ eV per unit cell. The optimized lattice constant of bulk ZGO was determined to be 8.334 Å.

To evaluate the work function of NO, CO, CO_2_, NO_2_, H_2_S, and O_3_ molecules adsorption on ZGO(111) surface, we employed a slab model consisting of 112 atoms with in-plane lattice dimensions of 11.85 Å × 11.85 Å. A vacuum spacing of at least 20 Å was introduced to prevent interactions between periodic images along the surface normal. The slab has two different terminations, named Zn-O and Ga-Zn-O. A previous study identified Ga-Zn-O surfaces as the lowest-energy surfaces, with an energy per unit area of 0.10 eV/Å^2^, reported by Jia et al. [18]. The top and side views of the considered NO, CO, CO_2_, NO_2_, H_2_S, and O_3_ adsorption sites on the Ga-Zn-O-terminated ZGO(111) surface are also shown in Figure 1b. A Γ-centered 3 × 3 × 1 Monkhorst-Pack *k*-point mesh was applied for Brillouin zone sampling in the density of states calculations. The entire supercell was fully relaxed until the residual forces on all atoms were below 0.001 eV/Å. The convergence of key computational parameters, including plane-wave cutoff energy, *k*-point sampling, slab thickness, and vacuum size, has been systematically examined and validated in our previous study on ZGO(111) surfaces [34]. In that work, convergence tests confirmed that a cutoff energy of 450 eV, a Γ-centered 2 × 2 × 1 *k*-point mesh for large surface supercells, and a vacuum spacing of approximately 44 Å yield well-converged total energies, work functions, and adsorption-related electronic properties. In the present study, the same surface orientation and comparable slab models are employed. A denser 3 × 3 × 1 Monkhorst–Pack *k*-point mesh is adopted to further ensure the convergence of adsorption energies and electronic properties. Therefore, the computational settings used here are consistent with, and more stringent than, those validated in our previous work.

**Figure 2:**
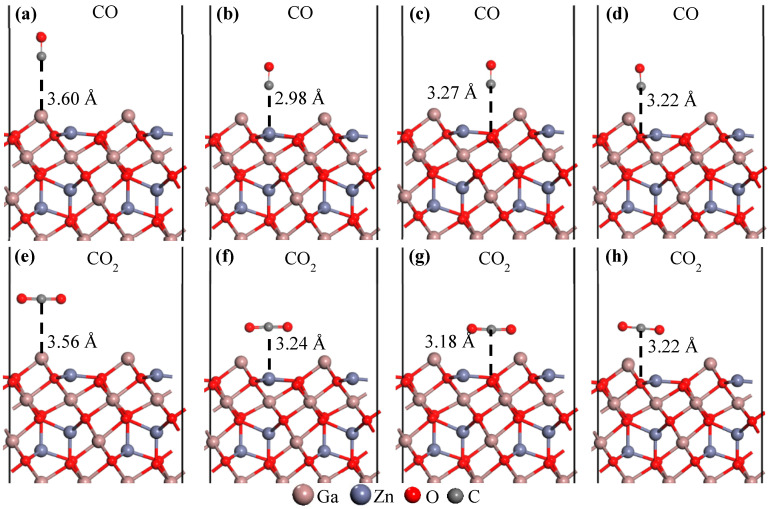
Adsorption behavior of CO, upper panel, and CO_2_, lower panel, molecules on the ZGO(111) surface at Ga_3c_ (**a**,**e**), Zn_3c_ (**b**,**f**), O_3c_ (**c**,**g**), and O_4c_ (**d**,**h**) sites. Atoms are represented by spheres: Ga (brown, large), Zn (gray, medium-sized), O (red, small), and C (gray, small).

**Figure 3:**
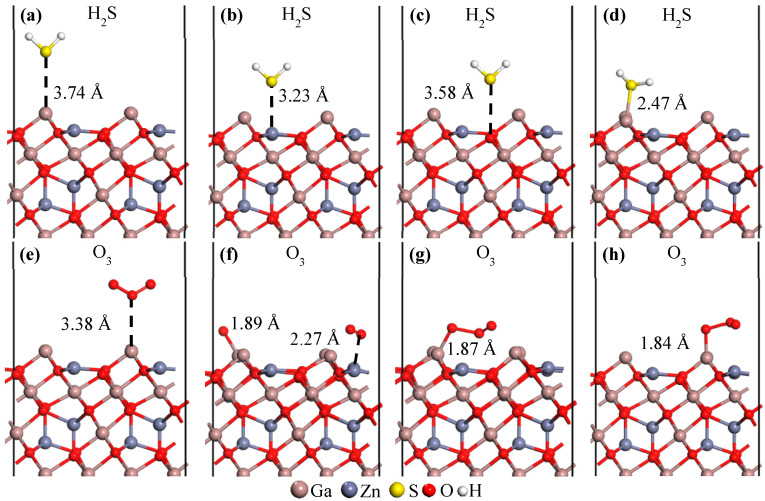
Adsorption behavior of H_2_S, upper panel, and O_3_, lower panel, molecules on the ZGO(111) surface at Ga_3*c*_ (**a**,**e**), Zn_3*c*_ (**b**,**f**), O_3*c*_ (**c**,**g**), and O_4*c*_ (**d**,**h**) sites. Atoms are represented by spheres: Ga (brown, large), Zn (gray, medium-sized), S (yellow, medium-sized), O (red, small), and H (white, small).

To determine the preferred adsorption sites of single NO, CO, CO_2_, NO_2_, H_2_S, and O_3_ molecules on the ZGO(111) surface, we labeled the surface atoms as Ga_3*c*_, Zn_3*c*_, O_3*c*_, and O_4*c*_ in the top and side views of Figure 1b. The final positions of the CO, CO_2_, H_2_S, and O_3_ molecules on the ZGO(111) surface are shown in Figure 2 and Figure 3. The initial distance between the adsorbed molecule and the ZGO(111) surface significantly influences the adsorption outcome. To ensure consistency in the calculations, the initial separation was set to the sum of the van der Waals radii of the interacting atoms. For instance, in the case of NO adsorbed on ZGO(111) Ga_3c_ site, the NO molecule is initially positioned directly above the Ga_3c_ site, with an initial distance of 3.42 Å (equal to the sum of the van der Waals radii of N and Ga).

Gas adsorption on the ZGO(111) surface modifies the work function change, ∆Φ, leading to a change in resistance between the target gas (*Rg*) and the reference gas (*Ra*). Usually, sensitivity (S) is defined as *Ra*/*Rg* for reducing gases or *Rg*/*Ra* for oxidizing gases, where *Ra* is the resistance of the gas sensor in the reference gas (usually air). Both *Ra* and *Rg* have a significant relationship with the surface reaction(s) taking place. The gas sensitivity is defined as *Rg*/*Ra*, with the work function shift expressed as follows [35]:

(1) ∆Φ= ∆X+kTln(Rg/Ra)

where ∆X is the electron affinity change, and *kT* is the thermal energy. The work function Φ is given by [36]:

(2) $$\Phi =E_{VAC}-{\;E}_{F}$$

with $E_{VAC}$ and $E_{F}$ denoting the vacuum and Fermi levels. Further, the adsorption energy ∆E is calculated as follows:

(3) $$∆E=E_{slab+molecule}-(E_{slab}+E_{molecule})$$

where $E_{slab+molecule}$, $E_{slab}$, and $E_{molecule}$ are the total energies of the adsorbed system, the clean ZGO(111) slab, and the isolated gas molecule, respectively. Both work function shifts and adsorption energies are considered in evaluating sensor sensitivity. Therefore, in the case of diatomic co-adsorption, we define the adsorption energy of the system as follows:

(4) $$∆E=E_{slab+X+Y\;}-(E_{X}+E_{Y}+E_{slab})$$

where $E_{slab+X+Y\;}$ is the total energy of the slab with both adsorbates *X* and *Y* molecule co-adsorbed, $E_{X}$ and $E_{Y}$ represent the total energies with only adsorbate *X* or *Y*, respectively, and $E_{slab}$ denotes the total energy of the clean slab. The lowest-energy single-molecule adsorption geometries used in this work were adopted from our previously validated systematic studies on ZGO(111) [25,26,34,37]. Binary co-adsorption configurations were constructed by combining these reference geometries and fully relaxing the systems to identify energetically competitive states.

## 3. Results and Discussion

In Section 3.1, we first discuss the variations in structure, adsorption energy, and work function associated with the adsorption of single gas molecules, NO, CO, CO_2_, NO_2_, H_2_S, and O_3_, on the ZGO(111) surface. Subsequently, in Section 3.2, we further analyze the case of multiple gases co-adsorption on the ZGO(111) surface. In practical oxide-based sensing, reducing gases commonly include NH_3_ in addition to CO- and sulfur-containing species. In the present work, however, we restrict the reducing-gas representatives to CO and H_2_S so that the co-adsorption space remains tractable while still capturing two distinct reduction chemistries (C-based vs. S-based) on the ZnGa_2_O_4_(111) surface. Including NH_3_ in co-adsorption configurations would significantly expand the configurational space and is therefore beyond the scope of the present work. Nevertheless, the conceptual framework established here—based on adsorption-induced charge transfer, work-function modulation, and synergistic or compensating interactions—provides a consistent basis for analyzing such effects should NH_3_ be considered in future studies. During structural optimization, in addition to the four adsorption sites considered in the previous section, i.e., Ga_3c_, Zn_3c_, O_3c_, and O_4c_, the initial orientations of the adsorbed molecules were also considered, including parallel and vertical placements relative to the surface. It is well recognized that the optimized adsorption configuration in first-principles simulations is highly sensitive to the initial geometric parameters, including the gas-surface distance and molecular orientation. This inherent dependence remains a methodological challenge, as an exhaustive sampling of all possible adsorption sites on the ZGO(111) surface is computationally prohibitive. In this work, we mitigated this limitation by systematically testing multiple representative initial geometries; however, a fully comprehensive search lies beyond practical feasibility. A promising future direction is to integrate machine-learning-based structure-prediction or global-optimization algorithms to accelerate the exploration of adsorption configurations and further improve the reliability of multi-gas adsorption studies.

### 3.1. Single-Molecule Adsorption

According to their molecular geometry, the adsorbates were classified into two categories. For geometrically linear molecules such as NO, CO, and CO_2_, only two initial orientations were considered, that is, parallel and perpendicular to the ZGO(111) surface. In the vertical configuration, taking the NO molecule as an example, both possibilities, either the N atom or the O atom facing the surface, were taken into account. For geometrically nonlinear molecules, NO_2_, H_2_S, and O_3_, the initial placements are inherently more complex; however, they can still be broadly categorized into parallel and perpendicular orientations relative to the surface, combined with multiple configurations depending on which specific atom of the gas molecule is located closer to the adsorption site.

#### 3.1.1. NO, CO, CO_2_ Molecule Adsorption

In the present work, we first consider gas molecules with a linear geometry, namely NO, CO, and CO_2_. Based on their atomic composition and molecular geometry, three representative initial adsorption configurations can be identified. For example, taking NO adsorption at the Ga3c site, in the vertical orientation, the molecule may be arranged with either the N atom or the O atom closer to the surface Ga atom. In addition, there is a configuration in which the NO molecule is aligned parallel to the ZGO(111) surface, giving rise to three possible adsorption modes. Following structural optimization, the configuration with the lowest adsorption energy is selected as the most stable adsorption state. Based on this optimized structure, the work function is subsequently calculated, and the variation in work function before and after adsorption is analyzed. The corresponding results are summarized in Table 1.

We found that NO adsorption at the O_3*c*_ and O_4*c*_ sites is unstable. The NO molecule consistently migrated to form a bond with the Ga_3*c*_ site during structural optimization. This behavior may be attributed either to the limitations in structural optimization or to the intrinsic instability of these adsorption sites. Moreover, it is evident that only NO exhibits a negative adsorption energy. In contrast, the adsorption energies of CO and CO_2_ are positive, indicating that these molecules are less likely to adsorb on the ZGO(111) surface. It should be noted, however, that our theoretical calculations are performed at 0 K, while the operating temperature of ZGO-based gas sensors is typically near room temperature (~0.03 eV). Since the adsorption energies of CO and CO_2_ are on the order of 0.5 eV (Table 1), this discrepancy corresponds to one order of magnitude, suggesting that the additional kinetic energy imparted to the molecules by temperature may also be insufficient to induce gas adsorption. Previous reports also conclude that all metal oxide sensors inevitably suffer from selectivity issues in practical NO_2_-detection applications, especially in the presence of reducing gases such as H_2_, and CO. Most gas sensors can only work properly at much higher temperatures above 200 °C [13].

In adsorption scenarios, van der Waals interactions occur between gas molecules and the ZGO(111) surface. Therefore, when the post adsorption bond length is significantly shorter than the initial bond length, the adsorption is identified as chemisorption; otherwise, it is classified as physisorption. With a gas sensor based on a resistive layer, only processes involving charge transfer between the solid and the gas can be detected. Therefore, the performance for the chemisorption is better than that of physisorption. Figure 2 illustrates the adsorption configurations of CO and CO_2_ molecules on the ZGO(111) surface. For CO molecules, as well as for NO molecules, the vertical adsorption mode is energetically more favorable, with the C (or N) atom positioned closer to the ZGO(111) surface. In contrast, CO_2_ molecules are more stable in the horizontal configuration. This difference can be attributed to the linear geometry and symmetric charge distribution of CO_2_, which favor a parallel alignment with the surface, thereby reducing repulsive interactions and enhancing surface stability.

#### 3.1.2. NO_2_, H_2_S, O_3_ Molecule Adsorption

We discuss the adsorption behavior of geometrically more complex molecules, namely NO_2_, H_2_S, and O_3_, on the ZGO(111) surface. The corresponding computational results are summarized in Table 2. As shown in Table 2, the adsorption energies of NO_2_ and O_3_ are negative, whereas all other configurations exhibit positive values. Regarding work function variations, H_2_S adsorption consistently results in negative shifts, whereas adsorption of NO_2_ and O_3_ molecules generally induces positive changes.

These results suggest that NO_2_ and O_3_ interact more strongly with the ZGO(111) surface than CO and CO_2_, enabling chemisorption under certain configurations. For the O_3_ molecule, the selective stability at the Ga_3*c*_ site indicates site-dependent adsorption behavior, reflecting the localized nature of O–Ga interactions. The negative work-function shift observed for H_2_S indicates electron donation to the surface, consistent with its reducing nature. In contrast, the positive shifts induced by NO_2_ and O_3_ adsorption are attributed to their oxidizing character, which tends to withdraw electrons from the surface. These contrasting trends highlight the distinct charge-transfer mechanisms involved in the adsorption of reducing versus oxidizing gases on the ZGO(111) surface.

Let us conclude single-molecule adsorption. From Table 1 and Table 2, it becomes evident that the ZGO(111) surface exhibits markedly different responses to each adsorbed gas molecule. Variations in the work function, adsorption energy, and preferred adsorption site collectively illustrate the extent of charge transfer and adsorption strength, which, in turn, determine the surface’s sensing capability toward specific gases. Among all examined species, O_3_ and NO_2_ induce the most pronounced modifications to the electronic properties of ZGO(111). When adsorbed at the Ga_3_*c* site, both molecules significantly increase the surface work function, for example, O_3_ raising it to 5.14 eV and NO_2_ to 4.75 eV, accompanied by significant work-function changes (Δ*Φ* = 0.97 eV for O_3_ and 0.58 eV for NO_2_). Their strongly negative adsorption energies (e.g., −1.59 eV for O_3_ and −1.15 to −1.55 eV for NO_2_) further indicate highly stable adsorption with substantial electronic rearrangement, characteristic of strong chemisorption. These results suggest that ZGO(111) is particularly sensitive to oxidizing gases.

In contrast, NO, CO, and CO_2_ show considerably weaker interactions with the surface. NO leads to only minor increases in the work function at both Ga_3*c*_ and Zn_3*c*_ sites, with adsorption energies ranging from −0.96 eV to −0.14 eV. CO and CO_2_ also induce relatively small work-function changes, and their adsorption energies, generally between 0.4 and 0.8 eV, reflect weaker adsorption. Notably, CO decreases the work function at the Ga_3_c site, indicating a distinct charge-transfer mechanism compared with strongly oxidizing molecules. The influence of the adsorption site also reveals consistent trends. Oxygen-rich oxidizing molecules, such as O_3_ and NO_2_, preferentially adsorb at the Ga_3*c*_ site, where they exhibit the strongest binding and most considerable work-function variations. In contrast, more neutral molecules, including CO and CO_2_, show relatively small energy differences among adsorption sites, consistent with weaker, physisorption-like interactions. H_2_S displays a unique behavior: adsorption at the O_4*c*_ site produces a substantial decrease in the work function (Δ*Φ* = −1.21 eV), suggesting significant electron back-donation to the surface. We note that the H_2_S@O_4*c*_ configuration exhibits a small positive adsorption energy (+0.07 eV) but a large work-function decrease (Δ*Φ* = −1.21 eV) (Table 2). This apparent mismatch reflects the fact that adsorption energy quantifies thermodynamic stabilization at 0 K, whereas the work function is highly sensitive to the interfacial dipole and charge redistribution. Even a weakly bound adsorbate can induce a substantial surface dipole through its adsorption geometry and polarization, leading to a pronounced Δ*Φ*. Given that +0.07 eV is comparable to typical thermal energy scales under sensing conditions, such a configuration may contribute via dynamic adsorption–desorption equilibrium, and its electron-donating character can effectively compensate oxidizing-gas-induced electron withdrawal in mixed-gas environments.

Overall, these trends indicate that ZGO(111) is highly responsive to oxidizing gases such as O_3_ and NO_2_, owing to their strong adsorption energies and substantial impact on the surface electronic structure. Conversely, molecules such as NO, CO, CO_2_, and H_2_S interact more weakly and induce more minor work-function changes. Consequently, work-function–based sensing on ZGO(111) is expected to be most effective for detecting strongly oxidizing species, while weakly interacting gases may require higher concentrations or auxiliary detection mechanisms.

### 3.2. Double-Molecule Adsorption

J. J. Vélez and co-workers investigated the gas-sensing behavior of SnO_2_ nanofilm sensors under the co-adsorption of NO_2_ and CO [38]. Their results showed that when a negative gate voltage was applied, NO_2_ adsorption decreased, whereas CO_3_ adsorption increased. This phenomenon arises because the negative electric field increases the surface electron density, thereby reducing NO_2_’s electron-capturing ability and decreasing NO_2_ adsorption. Meanwhile, CO reacts with surface oxygen species (O_2_^−^) to form CO_2_, which subsequently combines with lattice oxygen to form CO_3_^2−^, thereby increasing CO_3_ adsorption. Furthermore, the study demonstrated that the sequence of NO_2_ and CO adsorption influences surface charge coverage, indicating competitive adsorption. Specifically, CO adsorption alters the adsorption sites and coverage of NO_2_, while the presence of NO_2_ affects the reaction rate of CO oxidation to CO_3_. These findings show that external electric-field modulation can effectively tune the selectivity of SnO_2_ sensors toward NO_2_ and CO, thereby improving both sensitivity and specificity.

Nitric oxide (NO) exhibits dual oxidative and reductive characteristics in metal-oxide-based sensing systems, depending on the oxide material, surface chemistry, and sensing mechanism involved. For ZnGa_2_O_4_-based sensors, existing experimental evidence indicates that NO predominantly acts as an oxidizing species, thereby withdrawing electrons from the conduction band and increasing electrical resistance. In contrast, NO has been reported to act as a reducing gas on other oxide systems, such as WO_3_/Co_3_O_4_ composites [28], highlighting its material-dependent redox behavior. In the present study, the oxidative or reductive nature of NO on ZnGa_2_O_4_(111) is assessed within a consistent theoretical framework based on adsorption-induced charge redistribution and work-function modulation. An increase in the surface work function is interpreted as indicating electron withdrawal from the surface, consistent with oxidizing behavior. Within this framework, NO adsorption at stable Ga_3*c*_ and Zn_3*c*_ sites on ZnGa_2_O_4_(111) exhibits oxidizing characteristics, consistent with available experimental observations. Although not all adsorption configurations are energetically stable, the overall trend supports classifying NO as predominantly oxidizing on ZnGa_2_O_4_ under the conditions considered.

ZGO and SnO_2_ have both emerged as popular gas-sensing materials in recent years; therefore, the co-adsorption of two gas molecules on the ZGO(111) surface is also a topic worthy of investigation. In the case of dual gas adsorption, the possible adsorption configurations become significantly more complex. Specifically, there are four potential adsorption sites, namely Ga_3*c*_, Zn_3*c*_, O_3*c*_, and O_4*c*_. Considering that two different gas molecules may occupy any of these sites, there are 16 possible site combinations. Furthermore, each gas molecule may adopt different adsorption orientations depending on its molecular geometry, leading to at least 144 possible configurations even in the simplest scenario. To reduce computational complexity, we initially modeled co-adsorption by placing both gas molecules at the same adsorption site, without considering the case of different sites. After structural optimization, however, some of the configurations evolved into displaced adsorption geometries.

Table 3 summarizes the work function changes, Δ*Φ*, and adsorption energies, ∆E, for all cases of binary gas adsorption on the ZGO(111) surface. We first focus on the adsorption energy. As shown in Table 3, the adsorption energies are negative in most cases. Although the results in Table 1 and Table 2 indicate that CO, CO_2_, and H_2_S exhibit positive adsorption energies in single gas adsorption, this does not necessarily remain true under binary gas adsorption. Nevertheless, in the cases of (CO, CO_2_) and (CO_2_, H_2_S) double molecules, the adsorption energies are still positive. In addition, since two different gases are simultaneously adsorbed on the ZGO(111) surface, our constructed surface model has dimensions of 11.85 Å × 11.85 Å, with an average center-to-center distance of approximately 6 Å between the two adsorbates. Generally, this distance is sufficiently large to neglect direct interactions between the adsorbed gases. Furthermore, when comparing the cases with the lowest adsorption energies, it is observed that in most situations, the Ga_3*c*_ site provides the most stable adsorption configuration, or at least one of the two gas molecules is adsorbed at the Ga_3*c*_ site. The only exceptions are the combinations (NO, CO_2_), (NO_2_, CO_2_), and (H_2_S, O_3_).

**Figure 4:**
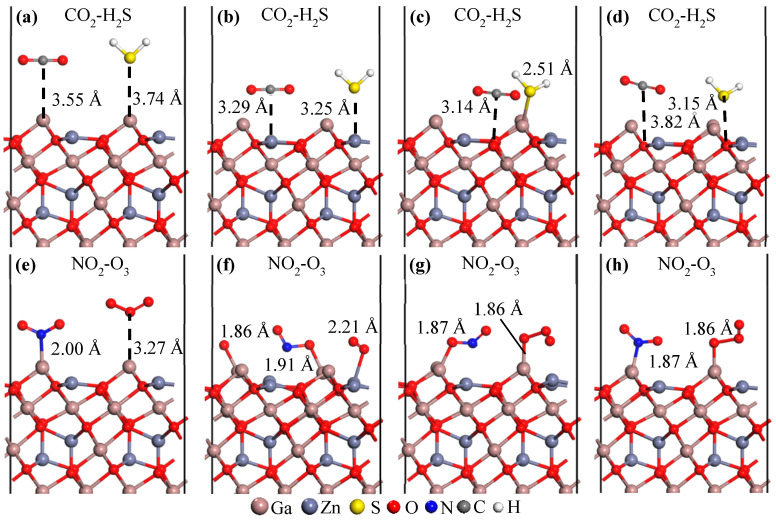
Adsorption behavior of (CO_2_, H_2_S), upper panel, and (NO_2_, O_3_), lower panel, molecules on the ZGO(111) surface at Ga_3*c*_ (**a**,**e**), Zn_3*c*_ (**b**,**f**), O_3*c*_ (**c**,**g**), and O_4*c*_ (**d**,**h**) sites. Atoms are represented by spheres: Ga (brown, large), Zn (gray, medium-sized), S (yellow, medium-sized), O (red, small), N (blue, small), C (gray, small), and H (white, small).

To better understand the co-adsorption behavior of two gas molecules on the ZGO(111) surface, the results of (CO_2_, H_2_S) and (NO_2_, O_3_) co-adsorption are presented in Figure 4. From left to right, Figure 4 shows the adsorption configurations at the Ga_3*c*_, Zn_3*c*_, O_3*c*_, and O_4*c*_ sites, respectively. It is evident that both CO_2_ and H_2_S exhibit physisorption behavior, whereas the (NO_2_, O_3_) pair clearly shows characteristics of chemisorption. Because chemisorption occurs, the adsorption energy of the (NO_2_, O_3_) gas is significantly lower. However, the variation in the work function does not become larger merely because of the smaller (i.e., more stable) adsorption energy. In the present study, such decoupling is clearly observed across several adsorption configurations, in which relatively weak adsorption still induces pronounced work-function shifts. This behavior indicates that effective modulation of the surface electronic structure—particularly interfacial charge redistribution and dipole formation—rather than adsorption stability alone, governs the sensing response. It is worth noting that the decoupling between adsorption stability and sensing response observed in this work is consistent with experimental findings reported for other multicomponent oxide sensing systems. Operando DRIFT studies, combined with electrical measurements, have demonstrated that pronounced sensor responses can arise from surface charge redistribution and dipole formation, even when the corresponding adsorption configurations are not the most energetically stable [39]. In particular, investigations on ternary oxide systems have shown that the nature of surface reaction strongly governs gas-induced electronic modulation intermediates and interfacial charge transfer, rather than adsorption energy alone. These experimental observations support the present theoretical interpretation that work-function modulation provides a more direct descriptor of sensing behavior than adsorption stability, particularly on complex oxide surfaces.

For a gas sensor, a larger change in the work function upon gas adsorption generally implies higher sensitivity toward the corresponding gas. Therefore, the absolute value of the change in work function can serve as an essential indicator for sensitivity evaluation. Table 3 records the variations in the system’s work function under binary gas adsorption. In general, both the type of adsorbed molecules and the adsorption sites significantly affect the magnitude of work function changes. As shown in Table 1 and Table 2, H_2_S exhibits the most significant work function variation of −1.21 eV. In contrast, the other gases, including NO, CO, CO_2_, NO_2_, and O_3_, display positive values of 0.33, 0.28, 0.24, 0.58, and 0.97 eV, respectively. If the work function change is primarily determined by the adsorbed gas type, the co-adsorption of H_2_S with other gases on the ZGO(111) surface may reduce the overall work function variation, thereby diminishing the sensor’s sensitivity.

This strong electron-donating character of H_2_S plays a decisive role in co-adsorption scenarios. When co-adsorbed with oxidizing gases such as NO or NO_2_, the electron donation from H_2_S partially compensates the electron-withdrawing effect of the oxidizing species, leading to pronounced non-additive behavior and severe cross-sensitivity. In contrast to weaker reductive gases such as CO, which exhibit nearly additive responses in co-adsorption, H_2_S significantly modifies the local adsorption geometry and electronic environment of the surface. As a result, H_2_S-containing gas pairs show work-function changes that deviate substantially from the simple superposition of single-gas contributions, highlighting the dominant compensatory role of H_2_S in mixed-gas environments.

**Figure 5:**
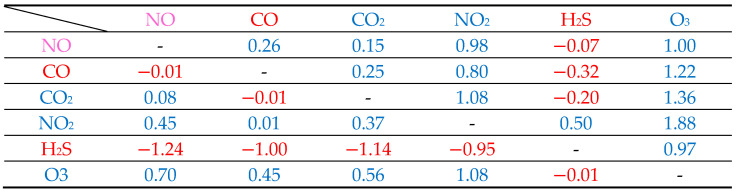
Synergistic interaction index matrix for binary gas co-adsorption on ZGO(111). Rows and columns correspond to the first and second gas in each pair, while diagonal entries marked “-“ denote single-gas adsorption for which a pairwise index is undefined. Red labels indicate reducing gases (CO and H_2_S), blue labels denote oxidizing gases (CO_2_, NO_2_, and O_3_), and magenta marks NO, which exhibits mixed redox character. Numerical values are color-coded by sign (blue: positive; red: negative). Using the diagonal as a visual divider, entries in the upper-right region represent pairs that enhance the work-function response, whereas those in the lower-left region indicate pairs that reduce it.

Figure 5 presents the synergistic interaction indices for all binary gas pairs co-adsorbed on ZGO(111). Positive indices indicate cooperative enhancement in the work-function shift, whereas negative values reflect compensatory interactions that suppress the net response. Strongly oxidizing gases, particularly O_3_ and NO_2_, exhibit pronounced synergy; the NO_2_–O_3_ pair yields the highest index (1.88), revealing a substantial amplification of the electronic perturbation beyond the sum of individual adsorptions. In contrast, H_2_S-containing pairs consistently display negative indices (e.g., NO-H_2_S: −1.24; CO-H_2_S: −1.00; CO_2_-H_2_S: −1.14), signifying substantial compensation due to the counteracting electron-donating character of H_2_S. These trends highlight that ZGO(111) is intrinsically more responsive to oxidizing species, whereas reducing gases such as H_2_S impose severe cross-sensitivity and must be carefully accounted for in mixed-gas sensing environments.

We finally conclude that the co-adsorption behavior of binary gas molecules on the ZGO(111) surface was systematically investigated to elucidate the interplay between adsorption configurations, electronic modulation, and adsorption energetics. The results reveal that most gas pairs preferentially occupy metal-coordinated surface sites, particularly the Ga_3*c*_ site, indicating that Ga atoms serve as the dominant reactive centers for charge transfer processes. Although initial configurations were distributed among Zn_3*c*_, O_3*c*_, and O_4*c*_ sites, the majority of co-adsorbed systems underwent structural rearrangement toward Ga_3*c*_ after relaxation, highlighting the strong interaction between adsorbates and surface Ga atoms. The adsorption energies (ΔE) demonstrate that co-adsorption on ZGO(111) is energetically favorable, with the majority of systems exhibiting strong binding (ΔE < −1.0 eV). Notably, pairs containing oxidizing species such as NO_2_ and O_3_ show the deepest adsorption energies, reaching up to ~−3.98 eV, followed by (NO, NO_2_) and (H_2_S, O_3_) systems with adsorption energies around −2.7 to −2.9 eV. These results suggest cooperative adsorption effects and strong chemical interactions, especially in systems involving highly reactive oxidizing gases. The significant stabilization of (H_2_S, O_3_) further indicates the presence of surface redox reactions, which contribute to the enhanced binding strength.

The co-adsorption induced substantial variations in the work function (Δ*Φ*), reflecting pronounced charge redistribution at the surface. Systematically, oxidizing gas pairs induced the largest positive Δ*Φ* shifts, consistent with electron withdrawal from the surface and enhanced *p*-type sensing responses. O_3_-containing systems exhibited the most prominent electronic modulation, with ΔΦ values exceeding +1.5 eV in several configurations. In contrast, systems involving reducing gases such as CO and H_2_S generally showed moderate or slightly negative Δ*Φ* shifts. However, co-adsorption with O_3_ still produced intensified electronic perturbation due to synergistic charge-exchange effects. Overall, the results demonstrate that ZGO(111) exhibits strong sensitivity toward oxidizing gases and enhanced signal amplification under co-adsorption conditions. The pronounced binding strength and work-function modulation particularly observed in NO_2_- and O_3_-based combinations indicate that ZGO is a promising candidate for multi-gas detection scenarios, especially in environments where competitive or cooperative adsorption phenomena are expected. When examining the work function changes under the most stable adsorption configurations (i.e., those with the lowest adsorption energies), an interesting observation emerges: the corresponding work function variations are not the largest. Although a larger change in the work function generally implies higher sensor sensitivity, this is not a strict requirement. As long as the work function change exceeds a certain threshold, the sensor can achieve the desired sensitivity. Therefore, the selectivity of ZGO toward different gas species should be interpreted in terms of response patterns and synergistic trends under co-adsorption, rather than as a unique single-gas signature. This perspective more accurately reflects realistic sensing environments in which multiple gases coexist.

**Figure 6:**
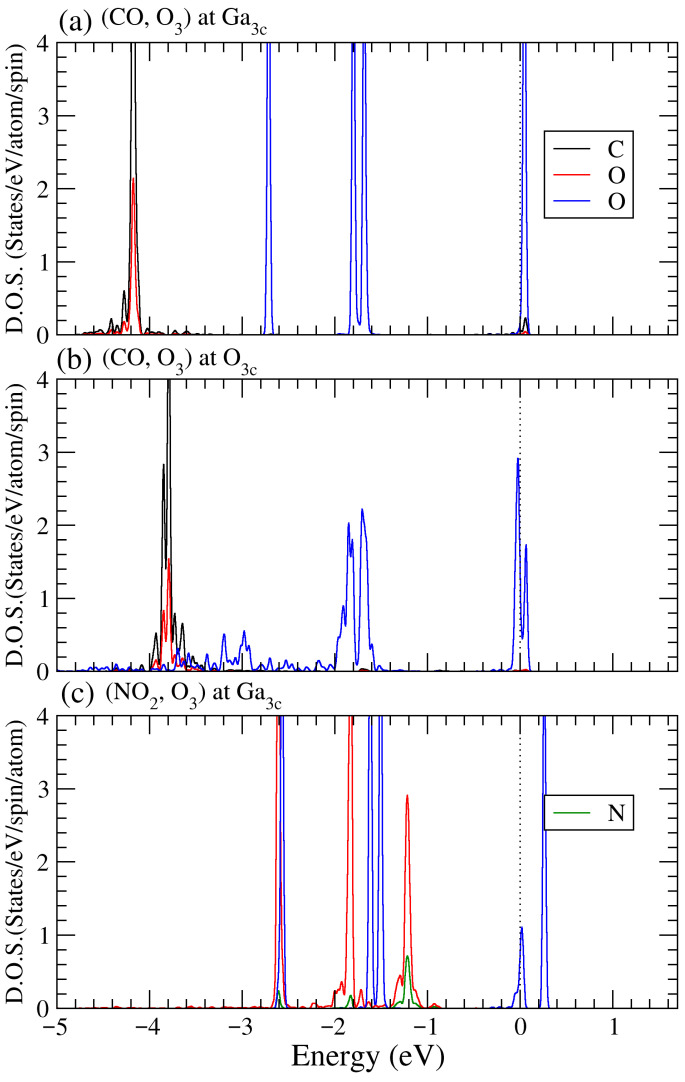
Orbital-resolved PDOS for representative binary co-adsorption systems on ZnGa_2_O_4_(111): (**a**) (CO, O_3_) adsorbed at the Ga_3*c*_ site, (**b**) (CO, O_3_) adsorbed at the O_3*c*_ site, and (**c**) (NO_2_, O_3_) adsorbed at the Ga_3*c*_ site. The vertical dashed line denotes the Fermi level (*E_F_* = 0 eV). Pronounced hybridization between O-2*p* (and N-2*p*) states of oxidizing gases and Ga-4*p* surface states below *E_F_* indicates strong charge transfer from the surface to the adsorbates, accounting for the observed work-function modulation.

To elucidate the microscopic origin of the work-function modulation induced by binary gas co-adsorption, orbital-resolved projected density of states (PDOS) analyses were performed for representative systems with distinct adsorption sites and sensing responses, as shown in Figure 6. For the (CO, O_3_) system adsorbed at the Ga_3*c*_ site, strong hybridization between O-2*p* orbitals of O_3_ and Ga-4*p* orbitals of the ZnGa_2_O_4_(111) surface is observed in the energy range of −1.5 to −3.0 eV below the Fermi level (*E_F_*). The formation of bonding states below *E_F_* indicates significant electron transfer from surface Ga atoms to the adsorbed O_3_ molecule, consistent with the substantial positive work-function shift. When the adsorption site is changed to O_3*c*_, the PDOS shows reduced orbital overlap and more delocalized features. Structural relaxation indicates that O_3_ still preferentially interacts with nearby Ga atoms, but partial dissociation and redistribution of electronic states weaken the effective charge-transfer pathway, resulting in a minor change in work function. In the case of (NO_2_, O_3_) co-adsorption, substantial overlap among N-2*p* and O-2*p* states of the adsorbates and Ga-4*p* surface states is observed over a broad energy range below *E_F_*. This cooperative orbital hybridization enhances electron depletion at the surface, thereby explaining the substantial increase in work function. These results confirm that Ga-coordinated sites play a dominant role in mediating charge transfer during multi-gas adsorption on ZGO(111).

It is noted that Figure 6a,b present the PDOS for (CO, O_3_) co-adsorption at the Ga_3*c*_ and O_3*c*_ sites, respectively. In both configurations, the CO-derived states are primarily located in the deep valence-band region around −4 eV, with no pronounced hybridized states within approximately ±0.5 eV of the Fermi level. This indicates that the interaction between CO and the ZGO(111) surface is dominated by dipole–surface electrostatic attraction and weak σ-type donation, rather than strong covalent bonding or π back-donation. In contrast, the O_3_-derived oxygen states exhibit pronounced features in the energy range from approximately −1.7 to −2.9 eV, indicating a much stronger perturbation of the surface electronic structure. As shown in Figure 6c, when NO_2_ and O_3_ are co-adsorbed at the Ga_3*c*_ site, the molecular states of both species are distributed closer to the valence-band maximum, reflecting substantial orbital hybridization and charge redistribution, which is consistent with the large work-function variations observed for this configuration. The preference of CO for oxygen-related surface sites can be attributed to its intrinsic molecular polarity and the electrostatic nature of the interaction. The CO molecule possesses a permanent dipole moment (Cδ^+^–Oδ^−^), which favors adsorption at surface oxygen sites with relatively higher electronegativity and localized negative charge. This interaction is primarily governed by dipole–surface electrostatic attraction and weak σ-type donation, rather than by strong covalent bonding or π back-donation, as evidenced by the absence of pronounced hybridized states near the Fermi level in the PDOS. Consequently, although the oxygen site provides the most favorable adsorption geometry for CO, the overall interaction remains weak in nature. These results indicate that adsorption on ZnGa_2_O_4_(111) spans from physisorption-dominated interactions to electronically activated adsorption, depending on the chemical nature of the analyte, rather than following a single universal adsorption mechanism.

In our previous studies, the adsorption behavior of single gas molecules on ZGO(111) surfaces was systematically investigated [25,26,34,37,40]. In contrast, the present work explicitly extends the scope to binary gas co-adsorption, aiming to bridge the gap between idealized single-gas models and more realistic sensing environments. The trends observed in this study are broadly consistent with established DFT literature on oxide-based gas sensors. Specifically, oxidizing gases, such as NO_2_ and O_3_, induce stronger adsorption, substantial electron withdrawal from the surface, and pronounced positive work-function shifts, whereas reducing gases, such as H_2_S, tend to donate electrons and partially compensate or reverse these effects. Similar behaviors have been widely reported in previous DFT studies of ZnO and SnO_2_ surfaces, in which oxidizing species act as electron acceptors and reducing species as electron donors. At the same time, the absolute magnitudes of adsorption energies and work-function changes obtained here may differ from those reported for other oxide materials or surface terminations. These deviations can be rationalized by the distinct chemical environment of ZGO(111), which features mixed cation sites (Zn and Ga) and unique coordination geometries. In particular, Ga-coordinated surface sites play a dominant role in mediating charge transfer, thereby enhancing sensitivity to strongly oxidizing gases relative to simpler binary oxides. Importantly, while many prior DFT investigations focus exclusively on single-gas adsorption, the present results demonstrate that binary co-adsorption can give rise to cooperative enhancement or compensating interactions that are absent in single-gas models. Consequently, the strong synergistic behavior observed for oxidizing–oxidizing gas pairs (e.g., NO_2_, O_3_), as well as the pronounced suppression induced by reducing gases, should be interpreted as a natural extension of established oxide sensing mechanisms to realistic multi-gas environments rather than as inconsistencies with previous studies.

## 4. Conclusions

Our theoretical calculations show that the work function changes in NO, CO, CO_2_, NO_2_, H_2_S, and O_3_ adsorbed on ZGO(111). For single-gas adsorption, H_2_S at the O_4_c site induced the largest work function change (−1.21 eV). In binary co-adsorption, NO_2_ and O_3_ at Ga_3_c exhibited the largest change (1.88 eV), highlighting enhanced electron transfer and sensing response. Co-adsorption of multiple oxidizing gases generally increases work function variations, improving sensitivity, whereas coexistence of oxidizing and reducing gases leads to smaller changes due to charge compensation, reducing sensor performance. These results provide guidance for designing ZGO-based gas sensors with improved selectivity and sensitivity.

## Figures and Tables

**Table 1:** Molecules (NO, CO, CO_2_), adsorption sites, the work function of the clean and gas-adsorbed surfaces ($\Phi_{S}$ and $\Phi_{S,gas}$), the resulting work function change (Δ*Φ*), and the adsorption energy (Δ*E*) on the ZGO(111) surface. “-” indicates not applicable (e.g., clean surface without adsorbates), while “---” denotes that no stable adsorption configuration was obtained after structural relaxation.

Molecule	Adsorption Site	$\Phi_{S,gas}$ (eV)	$\Phi_{S}$ (eV)	ΔΦ (eV)	ΔE (eV)
clean	-	-	4.17	-	-
NO	Ga_3*c*_	4.50		0.33	−0.96
	Zn_3*c*_	4.35		0.18	−0.14
	O_3*c*_	---		---	---
	O_4*c*_	---		---	---
CO	Ga_3*c*_	4.11		−0.06	0.53
	Zn_3*c*_	4.31		0.14	0.53
	O_3*c*_	4.45		0.28	0.80
	O_4*c*_	4.31		0.14	0.46
CO_2_	Ga_3*c*_	4.41		0.24	0.69
	Zn_3*c*_	4.24		0.07	0.65
	O_3*c*_	4.25		0.08	0.49
	O_4*c*_	4.32		0.15	0.76

**Table 2:** Molecule, adsorption site, work function ($\Phi_{S}$ and $\Phi_{S,gas}$), work function change (ΔΦ) and adsorption energy (ΔE) of the NO_2_, H_2_S, O_3_ molecule on ZGO(111) surface. “-“ indicates not applicable (e.g., clean surface without adsorbates), while “---” denotes that no stable adsorption configuration was obtained after structural relaxation.

Molecule	Adsorption Site	$\Phi_{S,gas}$ (eV)	$\Phi_{S}$ (eV)	ΔΦ (eV)	ΔE (eV)
clean	-	-	4.17	-	-
NO_2_	Ga_3*c*_	4.75		0.58	−1.15
	Zn_3*c*_	4.66		0.49	−1.55
	O_3*c*_	---		---	---
	O_4*c*_	---		---	---
H_2_S	Ga_3*c*_	3.92		−0.25	0.34
	Zn_3*c*_	3.88		−0.29	0.25
	O_3*c*_	4.03		−0.14	0.38
	O_4*c*_	2.96		−1.21	0.07
O_3_	Ga_3*c*_	5.14		0.97	−1.59
	Zn_3*c*_	4.89		0.72	−1.90
	O_3*c*_	---		---	---
	O_4*c*_	---		---	---

**Table 3:** Molecule, adsorption site, work function ($\Phi_{S}$ and $\Phi_{S,gas}$), work function change (ΔΦ) and adsorption energy (ΔE of the NO, CO, CO_2_, NO_2_, H_2_S, O_3_ molecule on ZGO(111) surface.

Molecule	Initial Adsorption Site	Final Adsorption Site		ΔΦ (eV)	ΔE (eV)
(*X*, *Y*)	-	*X*	*Y*	-	-
NO, CO	Ga_3*c*_	Ga_3*c*_	Ga_3*c*_	−0.01	−1.25
	Zn_3*c*_	Zn_3*c*_	Zn_3*c*_	0.10	−0.42
	O_3*c*_	O_4*c*_	O_3*c*_	0.26	−0.91
	O_4*c*_	O_3*c*_	O_4*c*_	0.17	−1.25
NO, CO_2_	Ga_3*c*_	Ga_3*c*_	Ga_3*c*_	0.13	−1.08
	Zn_3*c*_	Zn_3*c*_	Zn_3*c*_	0.08	−0.23
	O_3*c*_	O_4*c*_	O_3*c*_	0.08	−1.25
	O_4*c*_	Ga_3*c*_	O_4*c*_	0.15	−0.98
NO, NO_2_	Ga_3*c*_	Ga_3*c*_	Ga_3*c*_	0.98	−2.63
	Zn_3*c*_	Zn_3*c*_	Ga_3*c*_	0.45	−2.64
	O_3*c*_	Ga_3*c*_	Ga_3_*_c_*	0.59	−2.78
	O_4*c*_	O_4*c*_	Ga_3*c*_	0.79	−2.66
NO, H_2_S	Ga_3*c*_	Ga_3*c*_	Ga_3*c*_	−0.07	−1.39
	Zn_3*c*_	Zn_3*c*_	Zn_3*c*_	−1.24	−0.85
	O_3*c*_	O_3*c*_	Ga_3*c*_	−1.01	−1.75
	O_4*c*_	Ga_3*c*_	Ga_3*c*_	−0.93	−1.87
NO, O_3_	Ga_3*c*_	Ga_3*c*_	Ga_3*c*_	0.96	−0.49
	Zn_3*c*_	Zn_3*c*_	Zn_3*c*_	0.86	−0.16
	O_3*c*_	Ga_3*c*_	Ga_3*c*_	0.70	1.67
	O_4*c*_	Ga_3*c*_	Ga_3*c*_	1.00	1.51
NO_2_, CO	Ga_3*c*_	Ga_3*c*_	Ga_3*c*_	0.80	−1.23
	Zn_3*c*_	Zn_3*c*_	Zn_3*c*_	0.29	−1.86
	O_3*c*_	Ga_3*c*_	Ga_3*c*_	0.01	−1.94
	O_4*c*_	Ga_3*c*_	Ga_3*c*_	0.47	−1.82
NO_2_, CO_2_	Ga_3*c*_	Ga_3*c*_	Ga_3*c*_	1.08	−1.13
	Zn_3*c*_	Zn_3*c*_	Zn_3*c*_	0.37	−1.61
	O_3*c*_	Ga_3*c*_	O_4*c*_	0.48	−1.01
	O_4*c*_	Ga_3*c*_	O_4*c*_	0.82	−0.99
NO_2_, H_2_S	Ga_3*c*_	Ga_3*c*_	Ga_3*c*_	0.50	−1.47
	Zn_3*c*_	Ga_3*c*_	Zn_3*c*_	−0.02	−1.97
	O_3*c*_	Ga_3*c*_	Ga_3*c*_	−0.95	−1.87
	O_4*c*_	Ga_3*c*_	Ga_3*c*_	−0.49	−1.86
NO_2_, O_3_	Ga_3*c*_	Ga_3*c*_	Ga_3*c*_	1.88	−0.90
	Zn_3*c*_	Ga_3*c*_	Zn_3*c*_	1.08	−3.98
	O_3*c*_	Ga_3*c*_	Ga_3*c*_	1.33	−3.80
	O_4*c*_	Ga_3*c*_	Ga_3*c*_	1.56	−3.40
CO, CO_2_	Ga_3*c*_	Ga_3*c*_	Ga_3*c*_	0.02	0.52
	Zn_3*c*_	Zn_3*c*_	Zn_3*c*_	−0.01	0.46
	O_3*c*_	O_3*c*_	O_4*c*_	0.25	0.79
	O_4*c*_	O_4*c*_	O_4*c*_	0.04	0.27
CO, H_2_S	Ga_3*c*_	Ga_3*c*_	Ga_3*c*_	−0.49	0.20
	Zn_3*c*_	Zn_3*c*_	Zn_3*c*_	−0.32	0.11
	O_3*c*_	O_3*c*_	O_4*c*_	−1.00	0.02
	O_4*c*_	O_4*c*_	O_4*c*_	−0.79	−0.43
CO, O_3_	Ga_3*c*_	Ga_3*c*_	Ga_3*c*_	1.22	0.69
	Zn_3*c*_	Zn_3*c*_	Zn_3*c*_	0.64	−1.88
	O_3*c*_	O_3*c*_	Ga_3*c*_	1.00	−1.55
	O_4*c*_	Ga_3*c*_	Ga_3*c*_	0.45	−2.97
CO_2_, H_2_S	Ga_3*c*_	Ga_3*c*_	Ga_3*c*_	−0.20	0.32
	Zn_3*c*_	Zn_3*c*_	Zn_3*c*_	−0.37	0.24
	O_3*c*_	O_3*c*_	Ga_3*c*_	−1.14	0.04
	O_4*c*_	O_4*c*_	O_4*c*_	−1.05	0.18
CO_2_, O_3_	Ga_3*c*_	Ga_3*c*_	Ga_3*c*_	1.36	0.85
	Zn_3*c*_	Zn_3*c*_	Zn_3*c*_	0.56	−1.24
	O_3*c*_	O_3*c*_	Ga_3*c*_	0.88	−1.74
	O_4*c*_	O_4*c*_	Ga_3*c*_	0.92	−1.60
H_2_S, O_3_	Ga_3*c*_	Ga_3*c*_	Ga_3*c*_	0.97	0.41
	Zn_3*c*_	Zn_3*c*_	Zn_3*c*_	0.16	−2.89
	O_3*c*_	O_3*c*_	Ga_3*c*_	0.51	−2.22
	O_4*c*_	Ga_3*c*_	Ga_3*c*_	−0.01	−2.72

## Data Availability

The DFT datasets generated in this study are available from the corresponding author upon reasonable request.
